# Investigating predictive coding in younger and older children using MEG and a multi-feature auditory oddball paradigm

**DOI:** 10.1093/cercor/bhad054

**Published:** 2023-03-16

**Authors:** Hannah Rapaport, Robert A Seymour, Nicholas Benikos, Wei He, Elizabeth Pellicano, Jon Brock, Paul F Sowman

**Affiliations:** School of Psychological Sciences, 16 University Ave, Macquarie University, Sydney, 2109, Australia; Macquarie School of Education, 26 Wally’s Walk, Macquarie University, Sydney, 2109, Australia; Wellcome Centre for Human Neuroimaging, 12 Queen Square, London WC1N 3AR, United Kingdom; School of Psychological Sciences, 16 University Ave, Macquarie University, Sydney, 2109, Australia; School of Psychological Sciences, 16 University Ave, Macquarie University, Sydney, 2109, Australia; Macquarie School of Education, 26 Wally’s Walk, Macquarie University, Sydney, 2109, Australia; Department of Clinical, Educational and Health Psychology, 26 Bedford Way, University College London, London WC1E 6BT, United Kingdom; School of Psychological Sciences, 16 University Ave, Macquarie University, Sydney, 2109, Australia; School of Psychological Sciences, 16 University Ave, Macquarie University, Sydney, 2109, Australia

**Keywords:** Magnetoencephalography, Auditory, Mismatch field, Child

## Abstract

There is mounting evidence for predictive coding theory from computational, neuroimaging, and psychological research. However, there remains a lack of research exploring how predictive brain function develops across childhood. To address this gap, we used pediatric magnetoencephalography to record the evoked magnetic fields of 18 younger children (*M* = 4.1 years) and 19 older children (*M* = 6.2 years) as they listened to a 12-min auditory oddball paradigm. For each child, we computed a mismatch field “MMF”: an electrophysiological component that is widely interpreted as a neural signature of predictive coding. At the sensor level, the older children showed significantly larger MMF amplitudes relative to the younger children. At the source level, the older children showed a significantly larger MMF amplitude in the right inferior frontal gyrus relative to the younger children, *P* < 0.05. No differences were found in 2 other key regions (right primary auditory cortex and right superior temporal gyrus) thought to be involved in mismatch generation. These findings support the idea that predictive brain function develops during childhood, with increasing involvement of the frontal cortex in response to prediction errors. These findings contribute to a deeper understanding of the brain function underpinning child cognitive development.

## Introduction

Under predictive coding theory, the brain houses an internal, probabilistic, generative model which represents the statistical structure of the external world (Rao and Ballard 1999; Friston and Kiebel 2009; Hohwy 2013; Clark 2015). The brain uses this model to generate predictions about the most likely causes of incoming sensory signals, and tests its model by comparing its top-down prior predictions with bottom-up sensory signals from the world (Hohwy 2012). Predictions and sensory signals can be represented as probability distributions whereby the difference between the two represents the model’s “prediction error.” Prediction error is a useful learning signal as it indicates which sensory information the model failed to predict and hence, which information requires further processing at higher levels of the neural hierarchy (Dołęga and Dewhurst 2021). To reduce processing requirements, the brain strives to minimize prediction errors over time (Clark 2013).

An account of how children come to know about the world falls naturally from predictive coding principles (Scholl 2004; Gopnik 2012; Badcock et al. 2019). According to this view, infants are thought to begin life with a set of innately specified priors, rooted in the genes (Kersten and Yuille 2003). Following environmental stimulation, “innate priors” are updated through “perceptual inference” (Hohwy 2012) and subsequently called “empirical priors” (Kersten and Yuille 2003; Badcock et al. 2019). Thus, predictive coding takes advantage of both nature and nurture perspectives, allowing them to interact within a unifying framework. Iterative model updating should enable children to meet incoming sensory signals with increasingly accurate priors which should, in turn, result in fewer prediction errors, and hence greater confidence in the model’s predictions. Children’s priors may become increasingly precise as they gain experience in the world (Kayhan et al. 2019; Köster et al. 2020). As such, the posterior distribution (i.e. the perceptual experience; Hohwy 2012) would be gradually biased toward the increasingly narrow prior distribution (representing the brain’s stored knowledge), and away from the sensory signal distribution (Lucas et al. 2014).

Rapid model maturation across childhood may be underpinned by concurrent and significant neurophysiological changes. Indeed, the brain roughly quadruples in weight before age 6, by which time it has reached approximately 90% of its adult volume (Brown and Jernigan 2012). Furthermore, the efficiency of neuronal communication increases due to a prolonged period of synaptic pruning and myelination during childhood and adolescence (Blakemore and Choudhury 2006; Fischer and Bidell 2007; Santos and Noggle 2011). In particular, the protracted maturation of the prefrontal cortex (Huttenlocher and Dabholkar 1997)—a region thought to play a key role in extracting complex statistical regularities from incoming sensory signals (Basirat et al. 2014; Dürschmid et al. 2016; Jaffe-Dax et al. 2020)—may support increasingly sophisticated predictive brain function across development (Krogh et al. 2013). While a predictive coding account of neurocognitive development is supported by an extensive body of behavioral evidence (Gopnik and Wellman 2012; Köster et al. 2020), available neural evidence (Emberson et al. 2015, 2017, 2019a; Jaffe-Dax et al. 2020; Zhang and Emberson 2020) is in short supply —partly due to practical challenges associated with conducting functional brain recordings with young children (Barkovich et al. 2019). We sought to address this gap in the literature.

A popular method for testing predictive coding is to use electroencephalography (EEG) and/or magnetoencephalography (MEG) to record participants’ brain responses as they listen to an auditory oddball paradigm (Friston 2005; Heilbron and Chait 2018). These paradigms are comprised of high-probability “standard” and lower probability “deviant” stimuli (e.g. pure tones). Averaged evoked responses to the “standards” and “deviants” typically diverge between 0.1 and 0.25 s following stimulus onset, with the “deviant” showing a larger amplitude relative to the “standard” waveform (Näätänen et al. 2019b). This divergence—conventionally presented as a difference waveform—is the “mismatch negativity” (MMN; EEG literature) or the “mismatch field” (MMF; MEG literature; hereafter, “MMN/F” will be used when referring to both the MMN and MMF).

The MMN/F has been interpreted as a neural index of prediction error (Friston 2005), whereby the larger the evoked response amplitude, the larger the corresponding prediction error signal. With significant neurophysiological maturation during childhood (Huttenlocher and Dabholkar 1997; Blakemore and Choudhury 2006; Brown and Jernigan 2012), children’s brains may become increasingly proficient at extracting statistical regularities from the auditory input (Emberson et al. 2019b; Raviv and Arnon 2018). Consequently, older children may be able to form relatively precise priors of the upcoming stimuli, whereby the strongest prediction would be for the presentation of a high-probability “standard.” Thus, the presentation of a lower probability “deviant” may evoke a larger prediction error relative to that of a “standard.” This, in turn, would give rise to a relatively large MMF amplitude. By contrast, if younger participants are less proficient at extracting these statistical regularities, then they may form comparatively less-precise predictions for the upcoming stimuli. Thus, if younger children are less-precisely predicting the presentation of a standard, then the presentation of both high-probability “standards” and lower probability “deviants” may evoke similar degrees of prediction error, resulting in a relatively attenuated MMF.

Overall, one might expect a larger MMF amplitude in older relative to younger children, reflecting maturation of predictive brain function across development. However, previous EEG findings have been mixed, with studies reporting an increase (Oades et al. 1997; Bishop et al. 2011a; Chobert et al. 2014; Putkinen et al. 2014a; Putkinen et al. 2014b; Linnavalli et al. 2018), decrease (Csépe et al. 1992; Kraus et al. 1993a; Kraus et al. 1993b), or no difference (Kraus et al. 1999; Gomot et al. 2000; Shafer et al. 2000, 2010) in the MMN amplitude with age.

Furthermore, it appears that all previous studies employed EEG to investigate MMN maturation. Although MEG and EEG are similar in their millisecond temporal resolution, MEG offers advantages in terms of source-space reconstruction, as MEG magnetic fields are less susceptible to signal smearing and distortion compared with EEG electrical potentials (Baillet 2017). The scarcity of maturational MMF studies is likely due to the lack of availability of MEG systems—and pediatric MEG systems in particular—around the world (Azhari et al. 2020). Given the lack of child MEG-MMF studies, we currently know little about the early maturational changes in activity within the canonical MMN/F network. We sought to address this shortcoming in this study.

Here we aimed to test a predictive coding account of early neurocognitive development. To this end, we used pediatric MEG (Johnson et al. 2010; Rapaport et al. 2019) and a multi-feature auditory oddball paradigm, and measured MMF responses in younger (*M*_age_ = 4.1 years, range: 3.1–5.3) and older (*M*_age_ = 6.2 years, range: 5.6–6.9) children. We hypothesized that the older-relative-to-younger children would show a larger MMF amplitude, reflecting maturation of predictive brain function across development. Furthermore, we hypothesized that this maturation would be underpinned by increasing involvement of the frontal cortex in responding to prediction errors, as reflected by a significantly larger frontal-MMF in the older children.

## Materials and methods

### Participants

We recruited 60 children (aged 3 to 6 years) via the Macquarie University “Neuronauts” child research participation database. Of those, 3 children were unable to complete the MEG recording session due to anxiety (*n* = 2, aged 3.1 and 5.5 years) or falling asleep during the recording (*n* = 1, aged 3.1 years). Of the 57 collected datasets, 20 (35%) were excluded due to: (a) having a poor head position in relation to the MEG sensor array (*n* = 6; determined based on visual inspection); (b) excessive in-scanner head motion (based on real-time head motion tracking data) that exceeded an average of 10 mm over the recording (*n* = 4; *M*_age_ = 4.7 years, range: 3.2–5.8); (c) excessive in-scanner head motion (based on pre- and post-recording marker coil measurements as the real-time head motion tracking system was malfunctioning) that exceeded 5 mm (*n* = 5; *M*_age_ = 5.0, range: 3.2–7.0); (d) having more than 2 standard deviations above the mean for the number of interpolated channels (*n* = 4); and (e) missing triggers (*n* = 1). Thus, the final sample included 37 children (*M*_age_ = 5.2 years, SD = 1.2, range = 3.1–6.9 years; 20 females; 5 ambidextrous, 5 left-handed, based on parent report).

To investigate the (cross-sectional) developmental trajectory of the MMF, we performed a median age split—separating the data groups of younger children (*n* = 18; *M*_age_ = 4.1 years, SD = 0.9, range = 3.1–5.3 years; 8 females; 3 ambidextrous, 3 left-handed) and older children (*n* = 19; *M*_age_ = 6.2 years, SD = 0.4, range = 5.6–6.9 years; 12 females; 2 ambidextrous, 2 left-handed determined based on parent report). Normal hearing thresholds between 500 and 1,500 Hz were confirmed with pure-tone audiometric testing using an Otovation Amplitude T3 series audiometer (Otovation LLC, PA, United States). All children had parent-reported normal or corrected-to-normal vision and had no history of developmental disorders, epilepsy, brain injury, or language or speech impairment, as reported by parents. Children and their parents provided verbal and written informed consent, respectively, before the experiment. All procedures were approved by the Macquarie University Human Research Ethics Committee (reference: 5201600188). Families were paid 40 AUD for their participation and the children received a gift bag and certificate.

### Auditory oddball paradigm

Electrophysiological responses were measured as participants listened to a passive multi-feature auditory oddball paradigm (adapted from Näätänen et al. 2004). We chose to use the multi-feature over the traditional oddball paradigm as the former version offers advantages in terms of time efficiency—a quality which is of critical importance when testing young children who are generally less compliant than adults (Lovio et al. 2009). The multi-feature recording time is significantly reduced by presenting standards and deviants in an alternating pattern, as opposed to the traditional paradigm whereby the ratio of deviants to standards is between 1:7 and 1:10 (Petermann et al. 2009). No differences have been found between MMNs elicited by multi-feature compared with traditional oddball paradigms (Näätänen et al. 2004; Pakarinen et al. 2007). Furthermore, the multi-feature paradigm has been shown to reliably evoke mismatch responses in children (Petermann et al. 2009; Putkinen et al. 2012; Putkinen et al. 2014b).

**Fig. 1:**
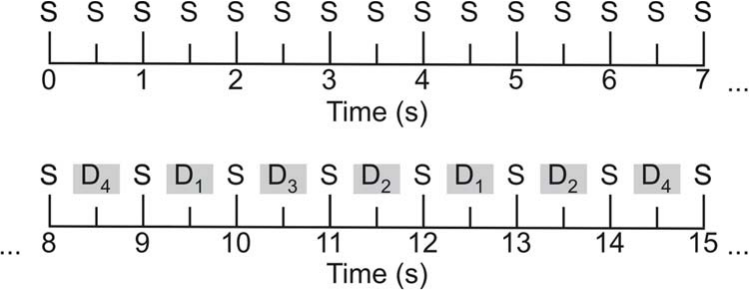
A schematic illustration of the multi-feature auditory oddball paradigm. The first 15 tones are “standards” (S) followed by alternating “standard” and 1 of 4 types of “deviant” (D) tones.

The paradigm consisted of 3 sequential stimulus blocks, each of which ran for approximately 4 min and consisted of 495 stimuli (0.5 s stimulus-onset-asynchrony). Block order was counterbalanced across participants. Each block began with the presentation of 15 “standard” stimuli followed by alternating “standard” and “deviant” stimuli (see Fig. 1 for a schematic diagram). The “standard” stimuli were sinusoidal harmonic tones which were 75 ms in duration (including 5 ms rise and fall times) and presented at a volume of 80 SPL and at a frequency of 550, 1,000, and 1,500 Hz (for each block, respectively). Each “standard” had a presentation probability of *P* = 0.5 (excluding the first 15 standards in each block).

The “deviant” stimuli differed from the “standards” in 1 of 4 acoustic features: (1) frequency (half were 10% higher [partials: 605, 1,100, 1,650 Hz] and half were 10% lower [partials: 495, 900, 1,350 Hz] than the standards), (2) intensity (half were 10% louder and half were 10% softer than the standards), (3) duration (the stimulus was presented for 25 ms with a silent 25 ms gap before the next stimulus), or (4) by having a silent gap in the middle of the stimulus (i.e. 7 ms removed from the middle of the stimulus tone, including 1 ms fall and rise times). Each of the 4 deviant subtypes had a presentation probability of *P* = 0.125. Deviants were presented in a pseudorandom order such that the same type of deviant could not be presented in succession. The paradigm was programmed and presented in MATLAB (Mathworks, Natick, MA, USA) using Psychtoolbox (Brainard 1997).

### MEG Acquisition

Electrophysiological data were acquired using a whole-head, supine, pediatric MEG system (Model PQ1064R-N2m, KIT, Kanazawa, Japan) housed in a magnetically shielded room (MSR; Fujihara Co. Ltd, Tokyo, Japan). The MEG sensor array contained 125 first-order axial gradiometers, each of which had a coil diameter of 15.5 mm and a baseline of 50 mm (see He et al. 2019 for further details). The dewar was designed to fit a maximum head circumference of 53.4 cm, accommodating the heads of more than 90% of 5-year-old Caucasian children (Johnson et al. 2010). All data were acquired at a sampling rate of 1,000 Hz and with an online bandpass filter of 0.03–200 Hz. To maximize the likelihood of obtaining high-quality data, we followed a child-friendly MEG testing protocol (see Rapaport et al. 2019).

Before the MEG recording, participants were fitted with a polyester cap containing 5 head position indicator (HPI) “marker” coils. A digitizer pen (Polhemus Fastrak, Colchester, USA) was used to record the locations of the HPI coils, as well as 3 fiducial points (the nasion and bilateral pre-auricular points) and 300–500 points from the scalp and face. During the MEG recording, participants listened to the multi-feature paradigm while watching a silent video of their choice. The auditory stimuli were presented via a 60 cm^2^ speaker (Panphonics SSH sound shower, Panphonics) positioned centrally at the foot of the MEG bed. The video was projected onto the MSR ceiling above the dewar. Participants’ head position in relation to the MEG sensor array was continuously monitored using a real-time marker coil tracking system (Oyama et al. 2012). Participants were accompanied by a researcher (and often a caregiver) during the MEG recording. The MEG procedures, including the setup and the recording, took approximately 45 min to complete.

### Data pre-processing

Following an inspection of the data, we removed 3 MEG channels that were consistently flat and noisy across participants for more than 10% of the recording (Gross et al. 2013). The following steps were performed using MEG160 (Yokogawa Electric Corporation and Eagle Technology Corporation, Tokyo, Japan). Environmental noise—estimated based on recordings from 3 reference magnetometers—was suppressed using a Time-Shift Principal Component Analysis (TSPCA) algorithm (de Cheveigné and Simon 2007; block width: 10,000 ms, 3 shifts). Data acquired with the real-time marker coil tracking system were used to correct for head motion artifacts (Knösche 2002; realignment conditions: sphere mesh = 321, prune ratio = 0.05). Head motion artifact correction could not be performed for 7 datasets that had missing or erroneous real-time coil tracking data for more than 10% of the recording. These 7 datasets were still included as head movement between the pre- and post-recording marker coil measurements did not exceed 5 mm.

Further pre-processing steps were performed in Matlab 2020a (MathWorths, Inc., Natick, MA, USA) using the Fieldtrip Toolbox (v20200213; Oostenveld et al. 2011). For each participant, the entire recording was high- and low-pass filtered at 0.1 and 40 Hz, respectively (using a onepass-zerophase firws filter with a Blackman window), and band-stop filtered to remove residual 50 Hz power-line contamination and its harmonics. Following visual inspection, segments of the recording containing artifacts (e.g. SQUID jumps and jaw clenches) were removed. Channels that contained a large number of these visually identified artifacts and/or were flat for more than 10% of the recording were interpolated to ensure that all participants had the same number of channels (Medvedovsky et al. 2007; NB. these removals were in addition to the 3 channels already rejected across the entire sample). An independent component analysis (ICA) was used to suppress eye blink artifacts: the raw recordings were high-pass filtered at 1 Hz to improve the ICA performance (Winkler et al. 2015) and then components with scalp distributions that corresponded to eye blinks were removed from the 0.1 Hz, pre-processed data. Up to one independent component was removed per participant.

The continuous data were epoched into segments of 0.5 s (0.1 s pre-, and 0.4 s post-stimulus onset). The first 15 epochs of each stimulus block were excluded from further analysis. Standard and deviant epochs were averaged across, respectively, to compute standard and deviant event-related fields (ERFs). It should be noted that we averaged across all 4 deviant conditions, as: (1) we sought to maximize the statistical power of our comparisons, and (2) we had no theoretical reason to analyze the 4 types of deviants separately. While the deviants differed in terms of their acoustic features, the constant feature that unites them is their pseudorandom occurrence (in contrast to the relatively predictable occurrence of the standards—see Fig. 1). The standard ERF was subtracted from the deviant ERF to produce an MMF.

### Sensor-level analysis

The sensor-level analysis involved 2 key steps performed in MNE-Python (Gramfort et al. 2013): (1) identifying a time-window of interest (TOI), and (2) constraining the age-related analysis to this time-window. We used a data-driven approach to identify a TOI. First, we reduced the multi-variate (channel x time) data from all 37 participants into a single time-course—achieved using an “Effect-Matched Spatial (EMS) filtering” approach (Schurger et al. 2013). EMS filtering involves estimating a spatial filter from the data itself, and then projecting the data through the spatial filter. EMS is superior to other data reduction methods (e.g. averaging a contiguous cluster of channels) as it accounts for the spatiotemporal evolution of the experimental effect across time and space, and weights the channels at each timepoint accordingly. EMS filtering was used to reduce the dimensionality of our data, and was orthogonal to our (between groups) hypothesis test.

To avoid circularity in the EMS filtering procedure, we employed 5-fold stratified cross-validation procedure (Huang et al. 2020; Engemann and King 2021). This analysis involved performing the following steps:

1. The data were normalized using z-scores.
2. A spatial filter was derived from 80% of the dataset, leaving out a different 20% for each repetition. The spatial filter was computed by subtracting the “standard” from the “deviant” epochs at each timepoint and channel. This produced a set of weightings (i.e. a spatial filter) that reflected the magnitude of the experimental effect (i.e. the MMF) over temporal and spatial dimensions.
3. The spatial filter was applied to the remaining 20% of the dataset by taking the product of the filter and the data at each time point and for each channel, and summing the results to render a single, spatially filtered time-course for the standard and deviant conditions, respectively.

The time-courses were then averaged across the 37 participants to form a single time-course for the “standard” and “deviant” conditions, respectively. Subsequently, we performed a nonparametric cluster-based permutation analysis (Maris and Oostenveld 2007) on the whole-group EMS filtered data to determine the time-course of the MMF effect. This permutation approach has been shown to adequately control the Type-I error rate for electrophysiological data. The analysis involved conducting one-sample *t*-tests at each time point to determine whether the MMF amplitude was significantly different from zero. We clustered samples whose *t*-values fell below a threshold corresponding to an alpha level of 0.05 (based on temporal proximity) and calculated cluster-level test statistics by taking the average of the *t*-values within each cluster. The data were then permuted 1,000 times, each time randomly shuffling the “standard” and “deviant” condition labels and recomputing the *t*-values. We constructed a permutation distribution from these random partition *t*-values. Finally, the significance of each cluster was determined using a threshold Monte-Carlo *p*-value.

To maximize statistical power, the time window of the significant cluster (identified in the analysis above) was used to constrain the age-related analysis. First, for each participant, we computed a mean MMF amplitude by taking the average of their EMS-filtered data within the TOI. Here the MMF was liberally defined as any significantly larger deviant-relative-to-standard amplitude within the TOI. Our definition stands in contrast to the definition of the classic adult MMN/F, which emerges soon after the second “N1” or “M2” component (Hari and Puce 2017, p. 265; Näätänen et al. 2019a, p. 53) and is visible between 0.1 and 0.25 s following stimulus onset (Näätänen et al. 2019b). We relied on this more liberal definition as the latency of the peak mismatch effect changes across childhood (Näätänen et al. 2019a) and it would therefore have been inappropriate to constrain our analysis to the classic adult MMN time window. It should be noted that prior pediatric auditory oddball studies have likewise used liberally defined time windows to extract MMN effects (e.g. 0.2–0.33 s in 6- to 7-year-olds, Lovio et al. 2009; 0.3–0.5 s in 4- to 12-year-olds, Partanen et al. 2013; 0.15–0.4 s in 5- to 7-year-olds, Petermann et al. 2009; 0.1–0.3 s in 9- to 13-year-olds, Putkinen et al. 2014a; 0.1–0.32 s in 4- to 10-year-olds, Shafer et al. 2000).

We compared mean MMF amplitudes between the younger and older children using a 2-sided permutation independent-samples *t*-test with 5,000 bootstrap samples. For each *p*-value, we performed 5,000 reshuffles of the age group labels using the DABEST (Data Analysis with Bootstrap-coupled ESTimation; Ho et al. 2019) open-source libraries for Python. Additionally, we used the Pearson correlation coefficient to investigate the relationship between mean MMF amplitude and age.

### Source-level analysis

To investigate how the neural network underpinning the MMF changes across age, we conducted source analysis on 6 predefined regions of interest (ROIs): bilateral primary auditory cortices (A1), bilateral superior temporal gyri (STG), and bilateral inferior frontal gyri (IFG; Garrido et al. 2007, 2008, 2009; Phillips et al. 2015). These ROIs were drawn from the adult literature as this is, to our knowledge, the first pediatric MEG study which has examined the neural sources underpinning MMN/F generation in typically developing children.

As the participants did not have individual structural MRI scans, we used the MRI Estimation for MEG Sourcespace toolbox (MEMES; Seymour 2018; https://github.com/Macquarie-MEG-Research/MEMES) to create surrogate structural MRIs. This procedure uses an Iterative Closest Point algorithm to match the participant’s digitized head shape information to an age-appropriate average MRI template (Neurodevelopmental MRI Database; Richards et al. 2016).

For each participant, the best-fitting template with the lowest objective registration (Gohel et al. 2017) was selected and used to create a cortical mesh and source grid and co-registered with the MEG sensor locations. A forward model (i.e. leadfield) was then computed using the cortical mesh as the volume conductor model. Source reconstruction was performed using a linearly constrained minimum variance (LCMV) beamformer (Van Veen et al. 1997), as implemented in Fieldtrip Toolbox (v20200213; Oostenveld et al. 2011). A spatial filter for each vertex in the source grid was computed using a free dipole orientation (Garrido et al. 2007). This step was performed separately for the “standard” and “deviant” conditions based on the covariance matrix calculated from the data combined across conditions. The spatial filters for all vertices within each ROI were combined into a single spatial filter by weighting voxels within the ROI according to their proximity to a centroid (Brookes et al. 2016). This centroid was defined as the voxel within the ROI that was nearest to all other voxels in the ROI (Douw et al. 2018). The sensor-level data were then right-multiplied by the spatial filters for each condition.

The final 3 analysis steps involved using the nonparametric cluster-based permutation approach (Maris and Oostenveld 2007). First, we used paired-samples *t-*tests to compare the “standard” and “deviant” amplitudes at each of the ROIs for all 37 children, permuting the condition labels 2,000 times. Second, we used independent-samples *t*-tests to compare the source-level (i) MMF (ii) standard, and (iii) deviant amplitudes between the groups of younger and older children, permuting the group labels (“younger” and “older”) 2,000 times. The experimental paradigm, data pre-processing and data analysis scripts are freely available on an Open Science Framework repository: https://osf.io/35q8n/.

## Results

### Sensor-level results

**Fig. 2:**
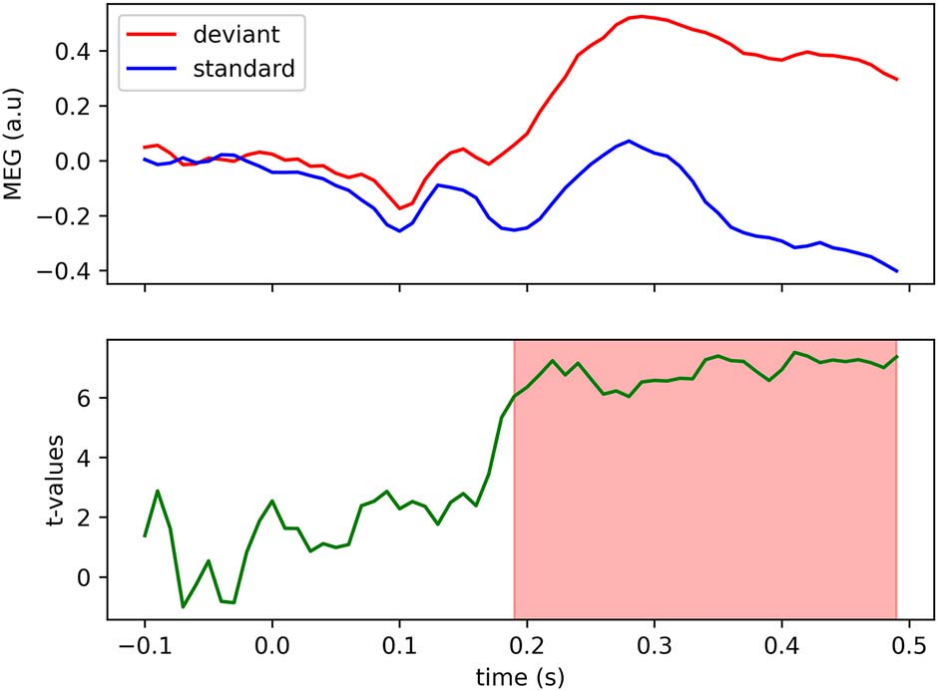
Top panel: Whole-group EMS filtered MEG data (a.u. = arbitrary units) for the standard (blue) and deviant (red) waveforms (*n* = 37). Bottom panel: Results from the cluster-based permutation test. The cluster of significant t-statistics is highlighted in red.

**Fig. 3:**
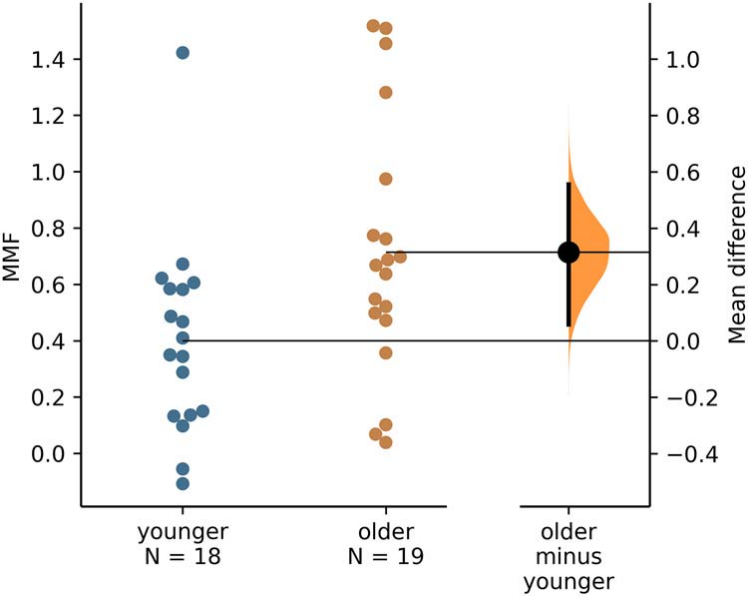
A Gardner–Altman estimation plot. Mean MMF amplitudes for all participants are plotted by age group on the 2 left-most axes. The effect size is presented as a bootstrap 95% confidence interval on the separate but aligned right-hand-side axis, and is displayed to the right of the data. The mean value of the older group is aligned with the effect size.

**Fig. 4:**
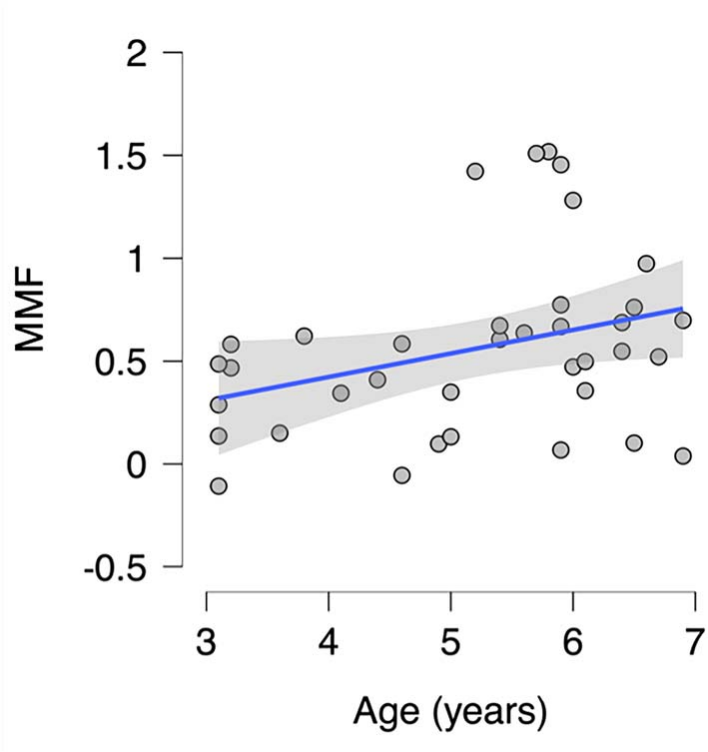
Scatterplot of the mean MMF responses and age for all participants (*n* = 37). The shaded area represents the 95% confidence interval.

Across all 37 children, we found a significantly larger deviant-relative-to-standard (i.e. MMF) amplitude between 0.19 and 0.49 s following stimulus onset (see Fig. 2). Results from a 2-sided permutation independent-samples *t*-test (constrained to 0.19–0.49 s) indicated that the mean MMF amplitudes were significantly larger in the older children (*n* = 19; *M* = 0.71, *SD* = 0.45) compared with the younger children (*n* = 18; *M* = 0.40, *SD* = 0.34), *P* = 0.027, Cohen’s *d* = 0.77 (95% CI for Cohen’s *d*: 0.03–1.4; see Fig. 3). A moderate, positive correlation of borderline significance was found between mean MMF amplitude and age (Pearson’s *r* = 0.33, *P* = 0.049; see Fig. 4).

### Source-level results

**Fig. 5:**
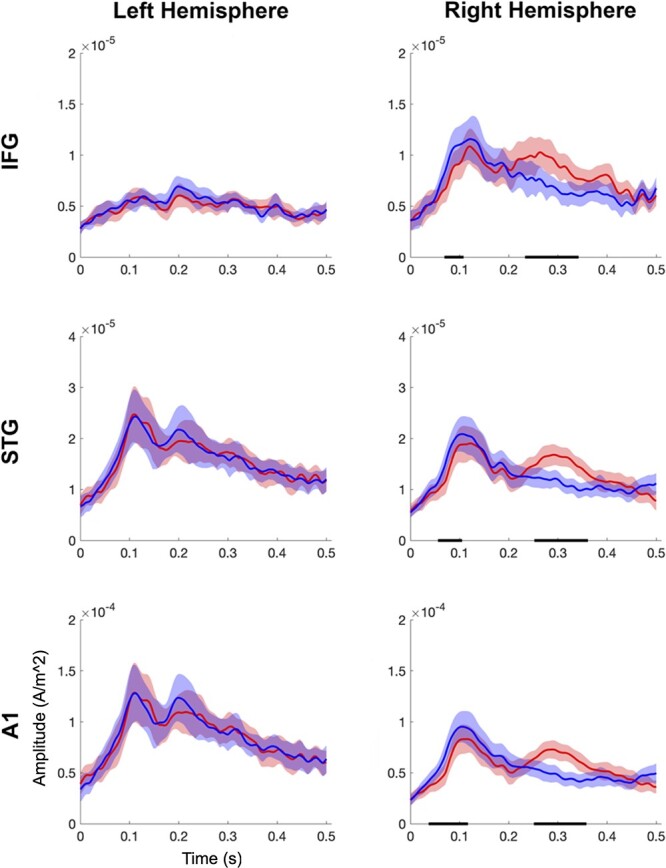
Whole-group source-level results. The 2 columns show the standard (blue) and deviant (red) ERF amplitudes (ampere per meter square) by time (s) for the left and right hemisphere ROIs, respectively. The shaded regions around each waveform represent 95% confidence intervals. The horizontal black line indicates clusters of significant differences (*P* < 0.05) between the standard and deviant ERF. Note the different y-axis scales.

**Fig. 6:**
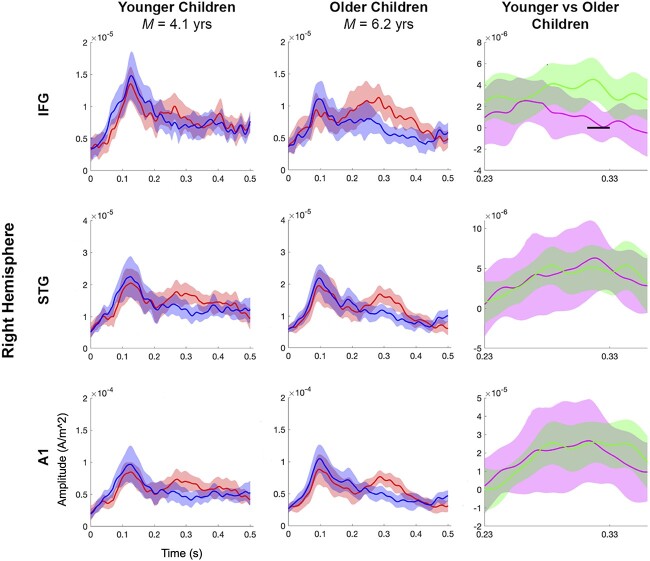
Source-level results for the younger and older children. The first 2 columns show the standard (blue) and deviant (red) ERF amplitudes (ampere per meter square) by time (s) for the younger and older children, respectively. The third column shows the MMF waveforms for the younger (pink) and older (green) children. The shaded regions around each waveform represent 95% confidence intervals. The horizontal black line indicates clusters of significant differences (*P* < 0.05) between the younger and older children’s MMF amplitudes. Note the different y-axis scales.

Across the entire sample, we found significantly larger MMF amplitudes, *P* < 0.05, in the 3 right-hemisphere ROIs: A1 (0.25–0.36 s), STG (0.25–0.36 s), and IFG (0.23–0.34 s; see Fig. 5). Hence, we constrained the subsequent age-related analysis to the right-hemisphere ROIs, and to the time window of 0.23 to 0.36 s. Consistent with our hypothesis, the older children showed a significantly larger MMF amplitude, *P* < 0.05, in the right-IFG (0.31 to 0.33 s) relative to the younger children (see Fig. 6). We found no significant differences between the younger and older children’s standard or deviant amplitudes in the same time window, *P* > 0.05.

## Discussion

This pediatric MEG study investigated the maturation of MMF between the ages of 3 and 6. Consistent with our hypotheses, we found that—at the sensor level—the older children (*M* = 6.2 years, range: 5.6–6.9) showed significantly larger MMF amplitudes relative to the younger children (*M* = 4.1 years, range: 3.1–5.3). Furthermore—at the source level—the older children showed a significantly larger MMF amplitude in the right IFG relative to the younger children. The larger MMF in older (relative to younger) children did not appear to be driven by either smaller or larger deviants alone, by rather a change in both directions, thereby giving rise to a significantly larger difference waveform across age. Our study is a first step toward measuring the early maturation of predictive brain function, with a focus on frontal regions like the IFG.

### The maturation of the MMF

The right-lateralized source-space findings are consistent with prior evidence that the classic, pure-tone-elicited MMN is most pronounced in the right hemisphere (Paavilainen et al. 1991). Furthermore, our finding of larger MMF amplitudes in older-relative-to-younger children is in line with the findings of previous EEG studies—both longitudinal (Chobert et al. 2014; Putkinen et al. 2014a; Putkinen et al. 2014b; Linnavalli et al. 2018) and cross-sectional (Oades et al. 1997; Bishop et al. 2011a)—which have similarly reported an increase in the MMN amplitude across childhood. Yet our results conflict with the findings of several other cross-sectional EEG studies, which have reported a decrease (Csépe et al. 1992; Kraus et al. 1993a; Kraus et al. 1993b) or no difference (Kraus et al. 1999; Gomot et al. 2000; Shafer et al. 2000, 2010) in the MMN amplitude with age.

One possible reason for the mixed findings regarding the maturation of the MMN/F may be related to variability in the methods used to calculate the MMN/F amplitude. Mean measures (calculated by taking the average amplitude across a time window) are generally considered to be superior to peak measures (calculated by locating the maximum amplitude within a given time window) as they are less susceptible to high-frequency noise distortions (Bishop 2007; Bishop et al. 2011b; Luck 2014a). Previous MMN studies that computed “mean” measures largely reported an amplitude increase with age (Bishop et al. 2011b; Chobert et al. 2014; Putkinen et al. 2014a; Putkinen et al. 2014b; Linnavalli et al. 2018), whereas those using “peak” measures reported either a decrease (Csépe et al. 1992; Kraus et al. 1993a; Kraus et al. 1993b) or no difference (Shafer et al. 2000, 2010) in the MMN amplitude with age. Given the problems associated with peak measures, the mean amplitude findings seem to be the most reliable and are consistent with the findings from this study.

The key finding of this study is that older children (*M*_age_ = 6.2 years) showed significantly larger MMF amplitudes in the right IFG relative to the younger children (*M*_age_ = 4.1 years). To our knowledge, this is the first pediatric MEG study which has examined the neural sources underpinning MMN/F generation in typically developing children. We cautiously interpret the current finding of larger MMF amplitudes in older children as reflecting the maturation of predictive brain function across childhood. Specifically, we suggest that with significant physiological maturation of the brain during childhood—and, the prolonged maturation of the frontal lobes (Huttenlocher and Dabholkar 1997)—children’s brains may become increasingly proficient at extracting statistical regularities from the stream of auditory input. Equipped with this statistical knowledge, children are then able to form increasingly precise priors of the upcoming stimuli, whereby the strongest prediction would be for the presentation of a high probability “standard.” The larger MMF difference waveform amplitude seen in the older children may reflect a larger prediction error response to the lower probability deviants (which may lie outside of the precise prior distribution), as well as a smaller error response to the higher probability standards (which may be precisely predicted). By contrast, the relatively attenuated MMF amplitude seen in the younger children may reflect—compared with the older children—a smaller prediction error to the deviants (which may lie within the broad prior distribution), as well as a larger response to the standards (which may be less precisely predicted). Furthermore, the results support the proposal that increasingly mature predictive brain function across development is underpinned by greater involvement of the frontal cortex in responding to prediction errors.

The notion that priors become increasingly precise with age is consistent with what we intuitively understand about the differences between adult and child perception (Lucas et al. 2014). Increasingly precise prior distributions across development would progressively bias the posterior distribution (i.e. the perceptual experience) toward the narrowing prior (i.e. the brain’s stored knowledge about the world) and away from incoming sensory signals. Furthermore, increasingly precise predictions about the world have an adaptive function, resulting in fewer prediction error signals and thus, reduced demands on neural bandwidth in the long run.

Conversely, having relatively broad priors in the early years of life would give rise to a perceptual experience that is biased toward incoming sensory signals from the world and less biased by prior knowledge. Furthermore, having imprecise, broad priors should yield more frequent prediction errors—driving more frequent model updating and thus boosting the learning rate. Indeed, it might be precisely because young children know less about the world that makes them more open to learning new information (Lucas et al. 2014).

### Alternative mechanistic accounts

Predictive coding theory provides a plausible account of the current findings. While alternative mechanistic accounts of the MMN/F have been put forward, these accounts can only account for MMN/F responses elicited by auditory oddball paradigms in which the “standards” are both: (a) acoustically identical to one another, and (b) repetitive in their occurrence. The “traditional” auditory oddball paradigm fits these criteria, involving the presentation of a stream of repetitive standard (S) stimuli that are infrequently interrupted by the presentation of a single deviant (D) stimulus (i.e. D-SSSD-SSSSS-D…).

Under the “sensory memory” account, a traditional MMN/F is generated when there is a detectable acoustic difference between the current input (e.g. a “deviant” stimulus) and a brief memory trace of the preceding input (e.g. a “standard” stimulus; Näätänen et al. 1978). Alternatively, under the “neuronal adaptation” account, neurons tuned to the “standards” become suppressed through repeated stimulation, whereas the rarer “deviant” stimuli activate non-adapted neurons (i.e. “fresh afferents”), resulting in an enhanced deviant-relative-to-standard evoked response (May and Tiitinen 2010). Finally, under predictive coding, the traditional MMN/F could reflect a relatively larger prediction error signal elicited in response to the lower probability “deviants” (*P* = 0.5/4) relative to the higher probability “standards” (*P* = 0.5). Thus, all 3 mechanistic accounts provide equally satisfying explanations of the traditional MMN/F.

Yet these sensory memory and neuronal adaptation accounts fail to explain MMN/Fs elicited by paradigms in which the “standard” stimuli are non-repetitive in their occurrence—such as the multi-feature paradigm implemented in this study (i.e. S-D_4_-S-D_2_-S-D_1_-S-D_4_-S-D_3…_). Under the “sensory memory” account, a change-detection mechanism would signal acoustic change on every trial, resulting in equally large evoked response amplitudes to all the stimuli and therefore, no mismatch effect. Likewise, under the neuronal adaptation account, the constant acoustic changes would not allow for habitation to any of the stimuli, again resulting in no mismatch effect.

### Study implications

Overall, the current neural findings offer preliminary support to the notion that predictive brain function improves across childhood. Our findings are consistent with a substantial body of behavioral evidence suggesting that young children learn the statistical regularities in their environment and use that statistical knowledge to generate predictions about future sensory events (Gopnik and Wellman 2012; Köster et al. 2020). The predictive coding account of childhood neurocognitive development represents a relatively new and radical way of conceptualizing early learning (Gopnik 2012).

This account may also have important implications for understanding the neurocognitive underpinnings of neurodevelopmental conditions that manifest in the early childhood years. For example, predictive coding accounts of autism have proposed that both the sensory and social characteristics of autistic people may be explained by divergent precision weighting of prediction errors (Brock 2012; Pellicano and Burr 2012; Friston et al. 2013; Pellicano 2013; van Boxtel and Lu 2013; Van de Cruys et al. 2013, 2014; Lawson et al. 2014, 2017). The current findings will serve as an important baseline for testing these predictive coding accounts of autism, as well as other neurodevelopmental conditions.

### Study limitations

It should be noted that the current findings are constrained by several limitations. The between-group analyses were conducted with relatively small samples (younger children: *n* = 18; older children: *n* = 19). While the effect sizes were large, studies with larger samples are needed to strengthen the present findings. With respect to the source analysis, our approach was constrained to 6 ROIs taken from Garrido et al. (2007, 2008, 2009): the bilateral primary auditory cortices (A1), STG, and IFG. We chose to constrain the analysis in this way in order to maximize the statistical power of the permutation approach (Luck 2014b). However, there is evidence of a more extensive MMN/F neural network beyond the frontotemporal regions, including generators in the parietal cortex (Celsis et al. 1999; Opitz et al. 1999; Schall et al. 2003; Molholm et al. 2005; Näätänen et al. 2019b). As such, important ROIs may have been left out of the current source analysis. Future research should attempt to investigate the neural generators more precisely beyond the regions investigated here with a considerably larger sample of participants (e.g. N = ≥ 100) using functional magnetic resonance imaging (fMRI) and/or MEG whole-brain source analyses.

## Conclusions

In conclusion, this study is the first to use MEG to investigate the maturation of the MMF during the childhood years. In line with our sensor-level hypotheses, we found larger MMF amplitudes in older (relative to younger) children. Furthermore, at the source-level, older children showed significantly larger MMF amplitudes in the right IFG relative to the younger children. We interpret these findings as preliminary support for the notion that predictive brain function matures across early development, and that this maturation is underpinned by increasing involvement of the frontal cortex in responding to prediction errors. These findings contribute to a deeper understanding of the brain function underpinning child cognitive development.

## Data Availability

The experimental paradigm, data pre-processing and data analysis scripts are freely available on an Open Science Framework repository: https://osf.io/35q8n/.

## CRediT authors statement

Hannah Rapaport: Methodology, Software, Formal analysis, Investigation, Data Curation, Writing – Original Draft, Writing – ReviewEditing, Visualization, Robert A. Seymour: Methodology, Software, Writing – ReviewEditing, Visualization, Nick Benikos: Investigation, Resources, Writing – ReviewEditing, Wei He: Methodology, Investigation, Supervision, Elizabeth Pellicano: Methodology, Writing – ReviewEditing, Supervision, Jon Brock: Conceptualisation, Writing – ReviewEditing, Paul F. Sowman: Conceptualization, Methodology, Software, Formal analysis, Writing – ReviewEditing, Visualization, Supervision, Funding acquisition.
